# Supplementary material for: Non-Invasive Iontophoretic Delivery of Cytochrome c to the Posterior Segment and Determination of Its Ocular Biodistribution
Source: Pharmaceutics. 2022 Aug 31;14(9):1832. doi: 10.3390/pharmaceutics14091832 (PMC9504550; doi:10.3390/pharmaceutics14091832)
Supplement: Supplementary file 1 [file pharmaceutics-14-01832-s001.zip › pharmaceutics-1839735-supplementary.pdf]

pharmaceutics

Supplementary Materials: Non-invasive Iontophoretic Delivery of Cytochrome c to the Posterior Segment and Determination of its Ocular Biodistribution

Laura Gisela González Iglesias[1], Siwar Messaoudi and Yogeshvar N. Kalia.

1. Development and validation of UHPLC-UV method for the quantification of Cytochrome c (Cyt c).

1.1. Sample preparation for analysis.

The complexity of the eye anatomy and its different compartments represent a challenge when it comes to sampling and ocular bioanalysis [28]. The most challenging matrices when it came to recovering Cyt c were the vitreous humor and the melanin containing tissues (iris, ciliary body, retina, and choroid). Ultrafiltration and HA degradation using hyaluronidase were performed to remove Cyt c from the vitreous humor and protein precipitation was used for the vitreous humor and the total ocular tissue matrix (matrix-obtained by combining the different tissues: cornea, aqueous humor, iris/ciliary body, retina, choroid, vitreous, and sclera).

1.1.1. Ultrafiltration

Amicon ultracentrifuge filters with a cut-off of 30 and 50 kDa were selected to separate Cyt c from the vitreous humour matrix. The cytochrome solution (100 µg/mL) was prepared using diluted vitreous humor matrix (aqueous dilution 1:1 w/w). 500 µL of Cyt c solution were centrifuged at 10,000 rpm for 5 min. At the end of the centrifugation period, the presence of Cyt c on the filter and its absence in the filtrate were evident (due to the red color) (**Figure S1**).

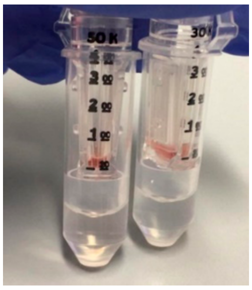

Figure S1. Ultrafiltration test using amicon ultracentrifuge filters (cut-off 30 and 50 kDa)

1.1.2. Hyaluronidase test

One of the most difficult samples to analyze was the vitreous humor as it is composed mostly of collagen and hyaluronic acid (28). This test was performed at 37 °C under gentle agitation. 1 mL of a 1 mg/mL hyaluronidase solution (PBS with BSA or in absence of BSA) was added to the vitreous humor sample (control) and to the vitreous sample with Cyt c (test solution). The samples were incubated for 45 minutes at 37 °C with gentle agitation. After the incubation period 2 mL of ACN was added to each sample (to simulate the mobile phase) followed by centrifuged at 5,000 rpm for 20 min. The supernatant was filtered through 0.22 µm filters and analyzed by UHPLC-UV. The test performed in the presence of BSA showed the best separation between the matrix and Cyt c, however the recovery was low (**Figure S2**).

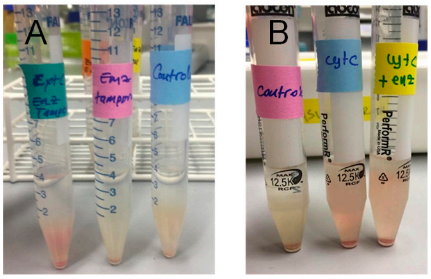

Figure S2. Hyaluronidase test, A) without BSA and B) in the presence of BSA.

1.1.3. Protein precipitation

The ocular tissue matrix was prepared using fresh pig eyes obtained from a local slaughterhouse (CARRE; Rolle, Switzerland). After cleaning the eyeballs, the corneas, aqueous humour, iris, choroid, retinas, vitreous bodies, and sclera were dissected and cut into small pieces, all the tissues were added in a glass vial containing 6.5 mL per eye of water and left to extract for 4 hours under magnetic stirring at room temperature. The ocular tissue matrix was used to simulate the most complex matrix from which Cyt c would be separated, a 200 µg/mL cytochrome c solution was prepared using the ocular tissue matrix to simulate the extraction samples. 1 mL of Cyt c solution was mixed with a) equal volume of acetonitrile, b) 100 µL of hydrochloric acid (1 M, pH=4.72) and c) equal volume of acetate buffer (pH 3). The final mixtures were gently agitated for 5 min and centrifuged at 6,000 rpm for 10 min to remove proteins and solid particles. The supernatant was filtered through 0.22 µm filters and analyzed by UHPLC-UV.

The addition of ACN produced the precipitation of the proteins present in the matrix but also of Cyt c. The best results were obtained when using an acid solution to precipitate the proteins present in the matrix (**Figure S3A**), the acid media decreased the interactions of Cyt c with the rest of the proteins allowing quantification of Cyt c in the supernatant (**Figure S3B**).

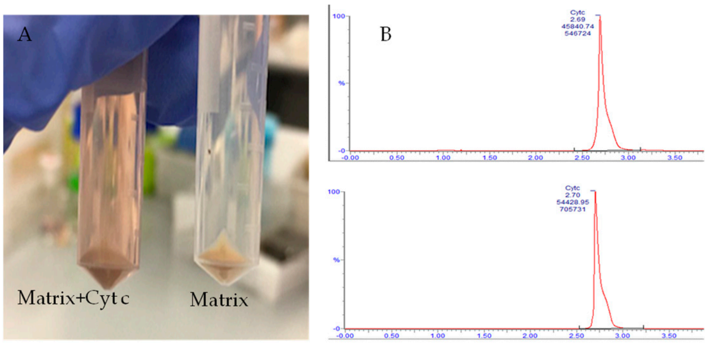

Figure S3. A) Matrix preparation in acid conditions, and B) quantification of Cyt c extracted in acid conditions (upper chromatogram-supernatant, superior chromatogram-control Cyt c solution).

1.2. *Chromatographic conditions*

The chromatographic separation of Cyt c from the ocular tissue matrix within a reasonable runtime was the most challenging part of the method development. Chromatographic conditions were optimized to obtain the best peak resolution, intensity, and shape. To achieve this, various conditions including mobile phase composition, flow rate, temperature and sample preparation were varied and optimized. The extraction of Cyt c from the different ocular tissues was performed using an acetate buffer at pH 3, which enabled precipitation of endogenous molecules present in the matrix without affecting Cyt c concentration. The best separation of Cyt c from ocular matrix under isocratic conditions was achieved using water+ 0.1 % TFA (A) and ACN: H_2O (95:5 v/v) + 0.1% TFA (B). However, the use of a gradient resulted in further improvement of the separation of Cyt c present in the biological matrix (**Table S1**).

Table S1. UPLC gradient for compound elution

Time (min)	Composition A (%)	Composition B (%)
0.0	80	20
8.5	50	50
9	80	20
12.5	80	20

Once the best chromatographic conditions were selected, the method was validated according to ICH guidelines with respect to selectivity and specificity, accuracy and precision, linearity, and sensitivity (lower limit of quantification, LLOQ).
The method specificity and matrix effect were evaluated using the ocular tissue matrix and the matrix obtained from individual tissues. The ocular tissue matrix solution was prepared using frozen enucleated eyes. Each tissue (except lens) was cut into small pieces and extracted with acetate buffer pH 3 (6.5 mL) and left for extraction for 4 h under agitation at room temperature, samples were centrifuged at 12,000 rpm for 10 min, then the supernatant was filtered using 0.22 μm cellulose acetate membrane syringe filters. For the individual tissue matrix, the tissues from 6 eyes were dissected and extracted with the corresponding extraction volume (**Table S2),** supernatants were collected and pooled (for each tissue), 6 eyes from different animals were used to prepare each ocular matrix.
The limit of detection (LOD) and quantification (LOQ) were established according to ICH Q2 (R1) guidelines [29].

Table S2. Extraction volume according to the tissue

**Tissue**	**Extraction volume Acetate buffer (mL)**
Cornea	0.5
Aqueous humor	0.5
Iris+ ciliary body	0.5
Retina	0.5
Choroid	0.5
Sclera	3
Vitreous humor	1

1.3. Specificity.

The method was specific for the quantification of Cyt c (t = 7 min) using a detection wavelength of 400 nm, and the peak was clearly separated from the solvent front and eye matrix. No interference or overlapping with the corneal extract components was detected at the retention time of 7 min for Cyt c. **Figure S4** presents the chromatograms obtained for Cyt c standard, buffer, ocular tissue matrix, individual tissue matrix (cornea, aqueous humor, retina, choroid, sclera, and vitreous humour), and Cyt c eye extraction samples

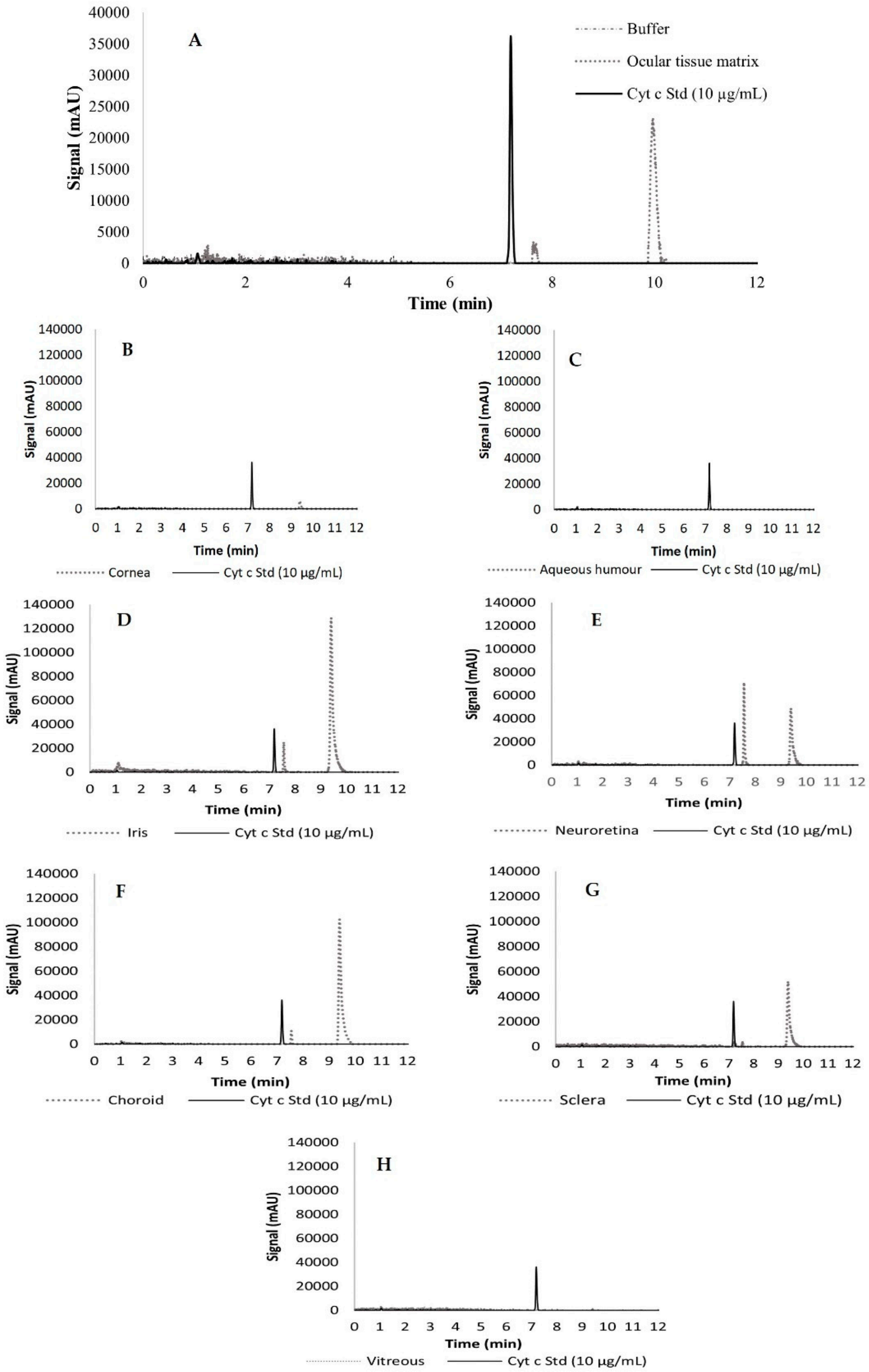

Figure S4. Chromatograms of (A) Cyt c standard (10 µg/mL), blank buffer and blank ocular tissues matrix, Chromatograms of Cyt c standard (10 µg/mL) and individual matrix of cornea (B), aqueous humor (C), iris (D), neuroretina (E), Choroid (F), sclera (G) and vitreous humor (H).

1.4. Linearity

The linearity of the method was asset by using a ten-point calibration. Good linearity was observed in a concentration range of 3-200 µg/mL with a R2 of 0.999 for Cyt c (Figure S5).

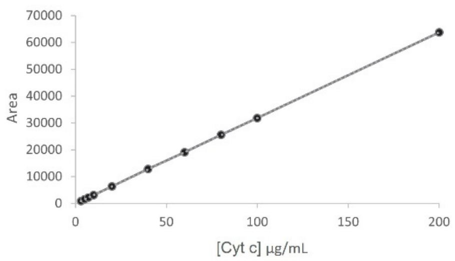

Figure S5. Example of calibration curve for Cyt c in ocular matrix

1.5. *Limit of detection and limit of quantification*

The limit of detection (LOD) and the limit of quantification (LOQ) were determined using the linear regression method and were 1 and 3 µg/ml, respectively.

1.6. *Precision and accuracy*

The intra-day precision and accuracy were evaluated by the analysis of standard solutions on the same day, whereas inter-day variability was tested on three different days. Results of accuracy and precision are listed in **Table S3** and were within the acceptance limits.

Table S3. Intra-and inter-day precision and accuracy for Cyt c quantification method

	Intra-day			Inter-day 1			Inter-day 2		
$[Cyt\ c]_{teo}$ (µg/ml)	$[Cyt\ c]_{meas}$ (µg/ml)	RSD (%) [a]	Recovery (%) [b]	$[Cyt\ c]_{meas}$ (µg/ml)	RSD (%) [a]	Recovery (%) [b]	$[Cyt\ c]_{meas}$ (µg/ml)	RSD (%) [a]	Recovery (%) [b]
200	200.72 ± 1.19	0.59	100.4	200.03± 0.80	0.4	99.8	202.69±1.95	1.0	101.3
80	79.37 ± 0.56	0.70	99.2	79.69 ± 1.10	1.4	100.9	80.38±0.38	0.5	100.5
20	20.18 ± 0.33	1.65	100.9	19.90 ± 0.14	0.7	100.0	20.31±0.33	1.6	101.8
5	5.34 ± 0.10	1.93	106.8	5.09 ± 0.18	3.6	101.3	5.44±0.12	2.2	106.2

[a] Precision = (SD/mean) *100, [b] Accuracy = (measured concentration/theoretical concentration) *100

References

28 Heinig K, Bertran E, Potter J, Fraier D. Ocular bioanalysis: challenges and advancements in recent years for these rare matrices. Bioanalysis. 2017;9(24):1997-2014.

29 Validation of Analytical Procedures: Text and Methodology Topic Q2 (R1), in: International Conference on Harmonisation of Technical Requirements for registration of Pharmaceuitcals for Human Use. [Internet]. 2005 [cited July 2018]. Available from: https://database.ich.org/sites/default/files/Q2%28R1%29%20Guideline.pdf
